# Menstrual Changes after COVID-19 Infection and COVID-19 Vaccination

**DOI:** 10.1155/2022/3199758

**Published:** 2022-10-27

**Authors:** Işılay Taşkaldıran, Emre Vuraloğlu, Yusuf Bozkuş, Özlem Turhan İyidir, Aslı Nar, Neslihan Başçıl Tütüncü

**Affiliations:** ^1^Department of Endocrinology and Metabolism, Faculty of Medicine, Başkent University, Ankara, Turkey; ^2^Department of Family Medicine, Faculty of Medicine, Başkent University, Ankara, Turkey

## Abstract

**Background:**

Several factors such as stress, depression, infection, and vaccination influenced the menstrual cycle in women during the coronavirus disease 2019 (COVID-19) pandemic. We investigated whether there were changes in the menstrual cycle in women after COVID-19 vaccination or infection and, if so, the nature of the change.

**Methods:**

This study was designed as a descriptive, cross-sectional study. A face-to-face survey was conducted among menstruating women aged 18–50 years from May 31 to July 31, 2022. Women were inquired about their first three menstrual cycles that occurred after COVID-19 infection or vaccination.

**Results:**

Of 241 women with COVID-19 infection, 86 (35.7%) mentioned that they experienced various changes in their menstrual patterns in the first three cycles after infection. Of 537 participants who received various COVID-19 vaccines, 82 (15.1%) stated that they experienced changes in their menstrual patterns after vaccination. The incidence of postvaccination menstrual change was higher in women who received Pfizer-BioNTech and Sinovac (CoronaVac) vaccines. Only 10.9% of women who reported a change in their menstrual pattern after vaccination or infection consulted a physician.

**Conclusion:**

COVID-19 infection and vaccination can affect the menstrual cycle in women. It is important to be aware of the menstrual changes after COVID-19 infection and vaccination and to warn and inform women about this issue.

## 1. Introduction

The COVID-19 pandemic, caused by SARS-CoV-2, has affected millions globally, resulting in serious mortality and morbidity. COVID-19 infection has caused serious health problems affecting all organs and systems, especially respiratory diseases [1]. One of the conditions affected by the COVID-19 pandemic is the menstrual cycle in women [2].

There are many factors that can affect menstrual cycles, such as stress, endocrine, gynecological, autoimmune, nutrition, genetics, infection, and changes in lifestyle. In many women, menstrual cycle irregularities were observed during the pandemic period. In recent studies, it is observed that SARS-CoV-2 infection itself, COVID-19 vaccines, and also stress in the pandemic may affect the menstrual cycle [2–4].

SARS-CoV-2 infection may affect the hypothalamic-pituitary-ovarian-endometrial axis, resulting in hypothalamic hypogonadism, which may cause temporary menstrual irregularities. In addition, ACE-2 receptors are widely expressed in the ovaries and endometrium, suggesting that SARS-CoV-2 infection may cause menstrual irregularities by directly affecting the ovaries and endometrium. Menstrual changes have also been reported following COVID-19 vaccination, which may be attributed to immunological processes [5–7].

Due to the insufficient and unclear data on COVID-19 vaccination and postinfection menstrual irregularities, warning and informing patients and society about the problems that can be expected remains insufficient, which increases social anxiety. In order to address this issue, we planned to evaluate the menstrual irregularities that can be observed after COVID-19 infection and COVID-19 vaccination by conducting a questionnaire on women of childbearing age.

## 2. Materials and Methods

This study was designed as a descriptive, cross-sectional study. A face-to-face survey was conducted with all menstruating women aged 18–50, who were admitted to our hospital between 31.5.2022 and 31.07.2022 or worked in our hospital. In the survey, questions were asked about their menstrual cycle for the first 3 cycles postinfection and post-COVID-19 vaccination, if they had a COVID-19 infection and/or had a COVID-19 vaccine. The study included women of reproductive age (18–50 years) alone. Pregnancy, lactation, menopause, and use of hormonal contraceptives were the exclusion criteria of the study.

The questionnaire comprised 17 questions and three parts: (1) identity and demographic questions, (2) COVID-19 infection history and 3 cycles of menstruation following infection, and (3) COVID-19 vaccination history and information about the next 3 cycles, if any.

The menstrual cycle in women was analyzed from three aspects: length of the menstrual cycle, duration of the period, and the amount of bleeding. The length of the menstrual cycle was classified as short (<24 days), normal, and late (>35 days). The duration of the period was classified as short (<3 days), normal, and long (>7 days). The amount of bleeding was classified as light, normal, and heavy. In addition, participants were asked if they experienced intermenstrual bleeding.

### 2.1. Statistical Analysis

SPSS 16.0 (IBM, Chicago, USA) software was used to analyze the data. When evaluating the study data, descriptive statistical methods (mean, frequency, and percentage) were used.

### 2.2. Ethical Consideration

This study was approved by the Başkent University of Medical Sciences Ethics Committee (Approval no. KA22/231).

## 3. Results

### 3.1. Participant Characteristics

The study included a total of 542 women, 487 (89.9%) of whom were single and 490 (90.4%) of whom were without children. The mean age was 23.34 years. The mean weight was 59. 54 kg, and the mean body mass index (BMI) was 21. 73 kg/m^2^. There were 439 (81%) participants without any chronic disease. Twenty-four (4.8%) participants had thyroid disease, and 21 (3.9%) were using thyroid drugs (levothyroxine or antithyroid drug). There were 52 participants (9.7%) with a previous diagnosis of the polycystic ovarian syndrome (PCOS). COVID-19 infection was detected in 241 (44.5%) of the participants, and it was found that 537 (99.1%) participants were vaccinated with any one of the available COVID-19 vaccines (Table 1).

### 3.2. Menstrual Changes after COVID-19 Infection

A total of 241 (44.5%) participants had COVID-19 infection. Of the 241 participants who had COVID-19, 86 (35.7%) stated that a change occurred in their menstrual pattern in the first 3 cycles following infection (Table 2). The changes in the menstrual pattern were as follows: shorter cycle in 34 (14.1%) patients (early menstruation), delayed cycle in 42 (17.4%) patients (late menstruation), intermenstrual bleeding in 10 (4.1%) patients, heavier menstrual bleeding in 18 (7.4%) patients, lighter menstrual bleeding in 14 (5.8%) patients, shorter period in 7 (2.5%) patients, and longer period in 10 (4.1%) patients (Table 3).

### 3.3. Menstrual Changes after COVID-19 Vaccination

Of 542 participants, only 5 (0.9%) had never been vaccinated and 537 (99.1%) had been vaccinated with various COVID-19 vaccines. Of those vaccinated participants, 446 (82.3%) had been vaccinated with the Pfizer-BioNTech COVID-19 vaccine, 39 (7.1%) with the Sinovac (CoronaVac) COVID-19 vaccine, 51 (9.4%) with both Pfizer-BioNTech and Sinovac (CoronaVac) COVID-19 vaccines, and 1 with the AstraZeneca COVID-19 vaccine.

Of 537 vaccinated participants, 82 (15.1%) stated that they had changes in their menstrual pattern after vaccination (Table 2). When those with the complaint of changes in their menstruation pattern after vaccination (*n*: 82) were evaluated, it was found that 68 received the Pfizer-BioNTech vaccine, 12 received the Pfizer-BioNTech and Sinovac (CoronaVac) vaccine, and 2 received the Sinovac (CoronaVac) vaccine. Of the participants with a change in the menstrual pattern, 43.3% stated a change in their menstrual pattern occurred after the 2nd dose of vaccine, 33.3% after the 1st dose of vaccine, 21.7% after the 3rd dose of vaccine, and 1.7% after the 4th dose of vaccine. Of the participants with altered menstrual patterns, 74.2% stated that these menstrual pattern changes did not recur in the following vaccinations.

A total of 537 (99.1%) patients had received various COVID-19 vaccines. Of these patients, 82 (15.1%) stated that a change occurred in their menstrual pattern in the first 3 cycles following vaccination. The changes in the menstrual pattern were as follows: shorter cycle in 20 (3.7%) patients (early menstruation), delayed cycle in 31 (5.7%) patients (late menstruation), intermenstrual bleeding in 13 (2.4%) patients, heavier menstrual bleeding in 20 (3.7%) patients, lighter menstrual bleeding in 14 (2.6%) patients, shorter period in 13 (2.4%) patients, and longer period in 16 (2.9%) patients (Table 3).

Of the patients who reported a change in their menstruation patterns following either vaccination or infection, only 10.9% consulted a physician, whereas 89.1% did not consult any physician.

## 4. Discussion

In our study, 35.7% of those who had COVID-19 and 15.1% of those who had COVID-19 vaccine reported various menstrual irregularities. These irregularities differed among women. Some women complained of shortened or delayed menstrual cycles, while some complained of heavier or lighter bleeding. The altered menstruation pattern following COVID-19 infection or vaccination is a very current issue, and different results are reported from different countries and races [5, 8, 9].

In our study, the incidence of menstrual changes after COVID-19 infection was higher than that of menstrual changes after vaccination, at 35.7%. In the Arizona CoVHORT study, the incidence of menstrual changes after COVID-19 infection was 16%, and in another study, it was 37.3% among hospitalized patients. In another study, the incidence of menstrual changes following infection was 47.2% [5, 8, 9].

In our study, the change in the menstrual pattern after COVID-19 infection occurred in the form of a delayed cycle in 17.4% of the participants, shortened cycle in 14%, heavier bleeding in 7.4%, lighter bleeding in 5.8%, shorter period in 2.5%, and longer period in 4.1%. In the literature, following infection, a delayed cycle was reported by 19.6% of the patients, a shortened cycle was reported by 15.2%, heavier bleeding was reported by 33%, a longer period was reported by 11.4%, and a shorter period was reported by 6.3% [5]. The incidence and the nature of the changes in our study are in concordance with the literature.

There may be various mechanisms underlying the menstrual changes following COVID-19 infection. Stress can be listed among these underlying mechanisms. COVID-19 has been shown to be a cause of severe stress, anxiety, and depression [10]. Stress can lead to menstrual irregularities through hypothalamic-pituitary-gonadal (HPG) axis dysfunction and consequent ovulation dysfunction [11]. In addition, previous studies have shown that stress causes ovulation disorders and menstrual irregularities by disrupting the preovulatory LH surge [12].

One of the causes of menstrual irregularities is thought to be the effect of the virus itself. The SARS-CoV-2 virus makes its way into the cell by binding to the ACE2 (angiotensin-converting enzyme 2) receptor, previously thought to be found in the respiratory tract alone, but now known to be found also in the ovary and endometrium. ACE2 receptors in the ovary play an important role in follicle maturation and ovulation [13]. ACE2 receptors are also found in oocytes from immature rat ovaries. In post-COVID patients, anti-Müllerian hormone was reported to be decreased, a phenomenon that may be related to the presence of ACE2 receptors in antral follicles. SARS-CoV-2 can directly invade ovarian follicles using ACE2 receptors, leading to reduced ovarian reserve and menstrual disorders. The role of ACE2 receptors in the endometrium is manifested in the involvement of angiotensin II in the initiation of menstruation through vasoconstriction of the spiral artery and angiotensin II-mediated increase in the proliferation of uterine epithelial cells. Therefore, the interaction between SARS-CoV-2 and ACE2 receptors in the endometrium may be among the causes of postinfection menstrual changes [13–15].

Studies have shown that cytokines such as interleukin-6, interleukin-8, and tumor necrosis factor-alpha, which are the mediators of the inflammatory response in COVID-19, can trigger a procoagulant state, resulting in changes in the menstrual pattern and in the amount of bleeding following infection [13, 16].

In our study, the incidence of changes in the menstrual pattern following COVID-19 vaccination was 15%, particularly after the second dose (43.3%). Various changes were reported, the most observed change being delayed menstruation (late menstruation). When the incidence of postvaccination menstrual changes in the literature was analyzed, it was found that it varied between 22 and 66% and was higher than that observed in our patient population [7, 17, 18].

When those with complaints of changes in the menstrual pattern after vaccination (*n*: 82) were evaluated in terms of their vaccination, it was found that 68 of these participants received Pfizer-BioNTech, 12 received Pfizer-BioNTech and Sinovac (CoronaVac), and 2 received Sinovac (CoronaVac). In other words, 12 (23.5%) of those who received Pfizer-BioNTech and Sinovac (CoronaVac), 68 (15.2%) of those who received Pfizer-BioNTech alone, and 2 (5.1%) of those who received Sinovac (CoronaVac) experienced menstrual changes. This demonstrated that the incidence of the postvaccination menstruation change was higher in those who received Pfizer-BioNTech and Sinovac (CoronaVac) and in those who received Pfizer-BioNTech alone.

The mechanisms underlying the vaccine causing menstrual irregularities are not yet clear. However, immune-mediated vaccine-induced thrombocytopenia can be considered a possible cause. Vaccine-induced thrombocytopenia and related menstrual changes have also been reported following many vaccinations previously (e.g., measles-mumps-rubella, hepatitis A and B, diphtheria tetanus-acellular pertussis, chickenpox, and influenza) [19, 20]. Another hypothesis is that vaccines may cause a strong immune reaction and stress that can temporarily affect the hypothalamic-pituitary-ovarian axis [21].

In our study, the incidence of consulting a physician for menstrual irregularities following vaccination or infection was 10.9%. The incidence of receiving medical help was observed to be very low. The reasons for this can be listed as the fear of being admitted to a hospital during the pandemic or ignoring the menstrual irregularity problem.

Our study has some limitations. First, we did not question the participants about hospitalization for COVID-19 infection, and hospitalization status may also cause menstrual irregularities. Besides, anticoagulation is another important issue during and after COVID-19 infection. Since our participants were mostly young females, the hospitalization anticoagulation rates are expected to be lower. Also, the survey questions were subjective, and the questionnaire is a retrospective method of evaluation. Finally, we could not reach enough participants due to pandemic conditions.

## 5. Conclusions

This survey-based study revealed that COVID-19 infection and COVID-19 vaccination can affect the menstrual cycle in women. Further prospective studies should be performed to confirm these findings and to evaluate how long these menstrual irregularities last and fertility. Women are less likely to seek medical help for infection and postvaccination menstrual irregularities. It is important to be aware of the menstrual changes after COVID-19 infection and the COVID-19 vaccination and to warn and inform women about this issue. Being aware of this issue and raising awareness among women will also reduce the anxiety that may occur due to menstrual irregularities.

## Figures and Tables

**Table 1:** Characteristics of the participants.

Parameters	
Participant, *n*	542
Age (years), mean (SD)	23, 34
Age (years), range	18–50
Marital status, *n* (%)	
Single	487 (89.9%)
Married	55 (11.1%)
Number of children, *n* (%)	
0	490 (90.4%)
1	25 (4.6%)
≥2	27 (5.0%)
Do you have any chronic disease? *N* (%)	
Yes	103 (19%)
No	439 (81%)
Have you ever had a COVID-19 infection? *N* (%)	
Yes	241 (44.5%)
No	301 (55.5%)
Did you get a COVID-19 vaccine? *N* (%)	
Yes	537 (99.1%)
No	5 (0.9%)

Values are expressed as a mean ± standard deviation or a number (%).

**Table 2:** Incidence of menstrual changes.

	(%)
Following COVID-19 infection	35.7
Following all COVID-19 vaccinations	15.1
After Pfizer-BioNTech	15.2
After Pfizer-BioNTech and Sinovac (CoronaVac)	23.5
After Sinovac (CoronaVac)	5.1

Values are expressed as a mean ± standard deviation or a number (%).

**Table 3:** Menstrual changes after COVID-19 infection and COVID-19 vaccination.

Menstrual parameters	Postinfection (*n*: 241)	Postvaccination (*n*: 537)
Menstrual cycle		
Shortened	34 (14.1%)	20 (3.7%)
Delayed	42 (17.4%)	31 (5.7%)

Period duration		
Shorter	7 (2.5%)	13 (2.4%)
Longer	10 (4.1%)	16 (2.9%)

Menstrual bleeding		
Lighter	14 (5.8%)	14 (2.6%)
Heavier	18 (7.4%)	20 (3.7%)
Intermenstrual bleeding	10 (4.1%)	13 (2.4%)

Values are expressed as a mean ± standard deviation or a number (%).

## Data Availability

The data used to support the findings of this study are available from the corresponding author upon request.
